# Quinine and Castor Oil

**Published:** 1873-08

**Authors:** 


### QUININE AND CASTOR OIL.

THE intense bitter of quinine is greatly modified to some tastes by the addition of fresh, sweet milk—not sweetened milk.

Castor oil, as useful as it is disagreeable, may be taken without much inconvenience by pouring it in the centre of half a glass of cold water, then hold the nose, open the mouth, put the edge of the glass far back on the tongue, toss it up quickly and down it goes. The philosophy of it is that, by not drawing in any air, and by keeping the lips separate, and not allowing the tongue to come in contact with them, no taste is imparted.

